# Covid-19: When Species and Data Meet

**DOI:** 10.1007/s42438-020-00180-x

**Published:** 2020-08-13

**Authors:** Catherine Price

**Affiliations:** School of Environmental Sciences, University of East Anglia, Norwich Research Park, Norwich, NR4 7TJ UK

**Keywords:** Covid-19, Species meeting, More-than-human worlds, Data, Contact-tracing apps, Postdigital hybrid assemblage, Anthropocene

## Abstract

This article explores how species meet, in particular humans and the Covid-19 virus. It also draws attention to the digital world through the lens of contact-tracing apps. Here, I examine human-virus-data relations, with humans, Covid-19, and data meeting and intra-acting. This article examines what has led us to this situation with Covid-19 and the role data is currently playing. The article offers an answer to two questions. How do humans, Covid-19, and Covid-19 contact-tracing apps meet and intra-act? What are the social justice issues and problems associated with contact-tracing apps? This article examines how species meet and intra-act, as well as how the Anthropocene has contributed to the current situation. The article also discusses contact-tracing apps and what these apps mean for society. Finally, the article shows how entanglements are not only constrained to those which are multispecies but also stretch out to the digital. These postdigital hybrid assemblages enable the coming together of humans, biological-more-than-human-worlds, and the digital. Postdigital hybrid assemblages enable us to push beyond boundaries, helping us understand Covid-19 and its impacts on society. Hopefully, this discussion about the postdigital hybrid assemblage will contribute to discussions in the future, and long after Covid-19, about how we are living our lives, and who and what we are living our lives with.

## Introduction

This article explores how species meet, in particular humans and the Covid-19 virus. It also draws attention to the postdigital world using digital contact-tracing apps as an example. Postdigital is understood here as ‘hard to define; messy; unpredictable; digital and analog; technological and non-technological; biological and informational’ (Jandrić et al. 2018: 895).

Digital contact-tracing uses a device such as a smartphone as a proxy for people. It measures the proximity between smartphones and then uses it as a proxy to determine the contact between two or more people (Ada Lovelace Institute 2020). Smartphones function through complex interactions between hardware (chips, processors, storage, and antennas), operating systems (Google [owners of Android] or Apple), app stores (Google or Apple), platforms (analytical companies who collect and analyse data, and social media companies), and apps (Privacy International 2020). With Covid-19, the data collected by smartphones is analysed by a risk-scoring algorithm to determine whether a user or the public health authorities should be contacted if a user has come into contact with a person with Covid-19. The use of this technology by governments is to help address the global public health emergency.

Across the world, we find human-virus-data relations, with humans, Covid-19, and data meeting and intra-acting. This article examines what has led us to the situation we find ourselves in with Covid-19, as well as the role data is currently playing. The article offers an answer to two questions. How do humans, Covid-19, and Covid-19 contact-tracing apps meet and intra-act? What are the social justice issues and problems associated with contact-tracing apps? In the next section, I explain how species meet and intra-act, drawing on Donna Haraway’s work for species meeting, and Karen Barad’s work on intra-actions. I then discuss the relationship between the Anthropocene and Covid-19. The multispecies entanglements which are present on Earth are inextricably linked to Covid-19 and the current situation we find ourselves in. I then move on to examine data and the National Health Service (NHS) Covid-19 app. An overview of contact-tracing apps from around the world is provided along with a more detailed discussion about the NHS Covid-19 app. This section also examines the social justice issues associated with contact-tracing apps. In the final section and the conclusion, I discuss how multispecies entanglements come together with the postdigital. Here, I explain how the coming together of humans, biological-more-than-human-worlds, and the digital can be considered a postdigital hybrid assemblage.

## Species Meeting and Intra-acting

A useful starting point for thinking through human-virus-data relations is the work of Donna Haraway. Haraway’s work examines the relationships between humans and the more-than-human. As Haraway (2008: 31) suggests, ‘the basic story is simple: ever more complex life forms are the continual result of ever more intricate and multidirectional acts of association of and with other life forms’. Our more-than-human world reaches beyond our relationships with animals. Bacteria and viruses are also part of the ecosystems in which we belong. They have also played a role in human evolution. As Haraway (2008: 3) explains,

I love the fact that human genomes can be found in only about 10 percent of all the cells that occupy the mundane space I call my body; the other 90 percent of the cells are filled with the genomes of bacteria, fungi, protists, and such, some of which play in a symphony necessary to my being alive at all, and some of which are hitching a ride and doing the rest of me, of us, no harm.

Of course, many microbes do not cause humans or animals harm. However, coronavirus is causing us harm. Coronavirus in all likelihood originated in animals (most likely a pangolin), and scientists are working to establish its origins (Cyranoski 2020). Humans often like to see themselves as separate to animals, but Covid-19 illustrates that all species are part of the same ecosystems. This includes viruses.

A virus is an infectious agent which reproduces within the living cells of an organism. As Nasir et al. (2012) explain, a virus has RNA- or DNA-based genomes with single- and double-stranded nucleic acids. Viruses do not have functional transcription machinery, so in order to replicate, they require a host. A host can be a plant, animal, or fungi, and once infected, the virus is able to spread throughout a population. The origin of life and of viruses is contested, and although there have been disagreements, there is a consensus that viruses are the key contributors to the evolution of cells. According to Nasir et al. (2012), there are three general theories that explain the origin of viruses. Firstly, the virus-first hypothesis asserts that viruses existed before cells and are what contributed to cellular lifeforms. Secondly, the reduction hypothesis views viruses as reduced forms of parasitic organisms which originated from cells. Thirdly, the escape hypothesis proposes that viruses were once part of the genetic material in host cells but escaped and evolved. Although there is no agreement as to which of these hypotheses is correct, viruses have played an important role in evolution. As Coffin (2004) explains, human and chimpanzee DNA differs by only a few percent even though they diverged from their common ancestor approximately five million years ago. Viruses which infected the common ancestor can be found in both human and chimpanzee DNA. These viruses are known as fossil viruses. Work currently being undertaken shows how ‘fossil viruses are also illuminating human evolution. Scientists estimate that 8.3 percent of the human genome can be traced back to retrovirus infections. To put that in perspective, that’s seven times more DNA than is found in all the 20,000 protein-coding genes in the human genome’ (Zimmer 2010).

Having outlined what a virus is, I return to the work of Donna Haraway. Some of Haraway’s work has developed in a mutual interaction with Karen Barad. Haraway uses Barad’s terminology of intra-action in describing what we might otherwise name human-animal ‘interactions’. For Barad (2003: 815):

The notion of *intra-action* (in contrast to the usual ‘interaction,’ which presumes the prior existence of independent entities/relata) represents a profound conceptual shift. It is through specific agential intra-actions that the boundaries and properties of the ‘components’ of phenomena become determinate and that particular embodied concepts become meaningful. … In other words, relata do not pre-exist relations; rather, relata-within-phenomena emerge through specific intra-actions.

What becomes known is due to specific interactions or intra-actions. Barad (2003) goes on to explain how *knowing* is perceived to be a human trait. However, we should see *knowing* as part of the wider world, with intra-actions between humans and the more-than-human world. We learn reciprocally. As such, humans are not separate to the rest of the world. We are part of the world in which we live and we are now sharing our world with coronavirus. The virus-animal-human interaction which has occurred illustrates a further point made by Barad (2003: 817), in that ‘agential intra-actions are specific causal material enactments that may or may not involve “humans.” Indeed, it is through such practices that the differential boundaries between “humans” and “nonhumans,” “culture” and “nature,” the “social” and the “scientific” are constituted’. Barad’s work helps us to start thinking about multispecies entanglements. These intra-actions enable a *coming together* and *entanglements*.

Haraway views this coming together and entanglements as becoming with. *Becoming with* is to *become worldly*. Species meeting brings humans and the more-than-human world together (Haraway 2008), and in doing so, humans become aware of the multiple worlds in which they encounter, and which they are part of. Becoming with gives up the idea of human exceptionalism. Coming together and entanglements become possible once we see all of life operating on the same plane, and that all lives are connected.

By considering the nature, culture, social, and scientific aspects of our lives, we can use all of our capacities and powers to make a life which connects to as many different species as possible. This is important because we need to ensure we are treating the more-than-human world both ethically and sustainably (Braidotti 2019). If we do not value the more-than-human world, we risk overexploiting it. By connecting with different species, we are connecting with kin. For Haraway (2016), kin is more than our relationships with other humans. It also involves our relationships with the more-than-human world through accountabilities and obligations. Haraway (2016: 103) explains how ‘making kin and making kind (as category, care, relatives without ties by birth, lateral relatives, lots of other echoes) stretch the imagination and can change the story’. By this stretching of the imagination, we are able to see viruses as kin, albeit an unpleasant kin. And this enables us to see why viruses can become part of us. We need to think beyond the species we can see and turn our gaze to the microbes as well. The word kin conjures up images of an assemblage, so perhaps it is time we think of kin as being all that inhabits the earth, from the microbial to the blue whale. This making kin approach enables us to situate our understanding of human-virus relations in a process of coevolution. This has occurred on two levels with Covid-19. Firstly, the virus is likely to have spread from animal to human. Secondly, the virus is spreading from human to human. Thinking about the virus spreading from human to human means, we have to think of Covid-19 as kin.

## The Anthropocene and Covid-19

The era we now live in has been termed the Anthropocene. ‘The term *Anthropocene* suggests that the Earth has now left its natural geological epoch, the present interglacial state called the Holocene. Human activities have become so pervasive and profound that they rival the great forces of Nature and are pushing the Earth into planetary *terra incognita*’ (Steffen et al. 2007: 614, emphasis in original). The Anthropocene is illustrated by the alteration of the global carbon cycle, the escalating loss of biodiversity, and the worldwide fragmentation of forests.

This human disconnection from the world is troubling and concerning. However, before the Covid-19 pandemic, climate change and harm to the environment had started making its way onto government agendas and had become more prevalent in people’s consciousness, especially as extreme weather events and melting ice sheets are now daily occurrences on the planet. But as Haraway (2015) argues, climate change, biodiversity loss, toxic chemicals, and the depletion of water resources and other physical resources are all threatening to collapse the Earth’s systems. This is a problem because it is the interconnected webs of multispecies entanglements which sustain life on Earth. However, in the era of the Anthropocene, many of these webs are disentangling. We are entering Earth’s sixth mass extinction event (Braidotti 2019; Wright 2017). The sixth mass extinction event means we need to broaden accountability and acknowledge the harms we are causing to the more-than-human world. I believe this means acknowledging multispecies entanglements as ‘knots of contradictions’ (Braidotti 2019: 15). Whilst multispecies entanglements are needed to sustain life on Earth, humans are just as likely to exploit them. Covid-19 most likely arose because of the human exploitation of another species. Only when we acknowledge these contradictions, can we move forward in addressing the problems we are collectively facing. Whilst it may not be immediately obvious what the relationship is between the Anthropocene and Covid-19, the two are inextricably linked.

When considering the relationship between the Anthropocene and Covid-19, it is necessary to think of the multispecies entanglements which are present on Earth, and the presence of zoonotic diseases. Part of the problem in the resurgence of zoonotic and vector-borne diseases is due to humanity encroaching evermore on wildlife habitats. For example, the Amazon rainforest being stripped of vegetation to make way for livestock production. The more contact humans have with wild animals, the greater the risk of a zoonotic disease being transferred to humans. SARS, bird flu, MERS, Ebola, Zika, and Nipah have all emerged from animals. Animals are now seen as the incubators, reservoirs, and spreaders of disease and a risk to humanity (Lynteris 2019). However, this is not a new problem. Lynteris (2019: 8) describes how the ‘regime of prevention and hope came to an end with the dawn of the emerging infectious diseases framework in the early 1990s, when scientists began to focus on processes leading to new diseases, hitherto of non-human animals, infecting humans and to the ‘species-jump’ processes (so-called spillover)’. This has led to animals being framed as the enemies of humanity as opposed to victims who have had their land encroached upon by humans.

The spaces animals occupy are often called *natural* or *wild*. In the Anthropocene, these spaces need to be thought of as uncertain, dynamic, and changing (Lorimer 2010). When there are changing and uncertain spaces, there can also be the breaking of boundaries. When animal diseases break boundaries and spillover into humans, public concerns about nature and risk are heightened, and governments have to focus on resolving disease outbreaks as a public health issue (Enticott 2009). However, we should not be surprised at these spillover events, as animal welfare organisations have been attempting to bring the issue of disease transmission and the threat to human health to the fore for a number of years (O’Sullivan 2020).

Whilst coronavirus did spillover from animals into humans, this time it is not animals spreading the disease as with rats with the plague. This time, it is humans spreading coronavirus. We can no longer think of Covid-19 as being separate to us. It is now part of our lives for the foreseeable future. If we think of Covid-19 as separate to us, this leads us to consider the idea of viruses as predators, and viruses as humanity’s prey. As Lynteris (2019: 14) argues ‘on the one hand, microbes are seen as predators of humanity, who lurk and hide so as to better ambush their prey. And on the other hand, as the enduring metaphor of ‘virus hunters’ amply illustrates, microbes are also seen as humanity’s prey—which thus ‘hide’ to escape being caught and vanquished by us’. This statement appears problematic in terms of Covid-19 because of the virus being highly contagious. Covid-19 is very much in plain sight because humans are the carriers of the virus. In past disease outbreaks when animals were carriers of a particular disease, animals were culled. For example, with an outbreak of bird flu (H5N1) in Hong Kong in 1997, the entire poultry population of 1.5 million birds was destroyed within 3 days. This was to prevent further human exposure when it was found there was bird to human transmission of the disease (Webster and Hulse 2005). Here though, is why Donna Haraway’s argument about making kin is so important.

Firstly, there is the spillover event and the transmission of Covid-19 from animal to human. Because of this, the virus has become part of humans’ lived experiences. Secondly, we need to consider the use of animals in laboratory testing. Animal testing is problematic if we consider animals as kin because it poses a contradiction. If animals are kin, then we should not be exploiting them. However, as Braidotti (2019: 109) argues, humans are ‘defined as a species that monopolizes the right to access the bodies of all living entities’. Accessing the bodies of animals in laboratory testing is done to serve human interests by keeping humans healthy or alive. This perspective sees human lives as more important and valuable than animal lives. The argument for testing on laboratory animals is to ensure new vaccines and drugs are safe before human clinical trials begin (Rowlands 2002). In the search for a vaccine for Covid-19, mice, monkeys, hamsters, rats, ducks, pigs, chickens, cats, dogs, and ferrets are being used as test subjects (Cohen 2020; O’Sullivan 2020). For Peggs (2011), experimentation can be conducted on animals because of human primacy identity politics. This type of politics encourages and maintains the human assumption of superiority over the more-than-human world. It is this human superiority which enables animals to be regarded as a resource to be exploited. As a resource, an animal can be described as an object which is subjected to an experiment as opposed to being an actor in an experiment (Adams 2018). As an object, animals have no interests or legal rights unlike a human actor. What we have to remember is that the ‘animal involved is likely to suffer considerably during the experiment, and will almost certainly be killed after the experiment’ (Rowlands 2002: 125).

Contesting and disputing the effectiveness of animal testing are not straightforward. If the legitimacy of animal testing is challenged, then opponents often face the criticism of being anti-science and opposing advances in science (Adams 2018). Covid-19 leaves us in a conundrum. Covid-19 as a human health problem emerged because of human exploitation of animals. To solve this health problem through animal testing means further exploitation of animals (O’Sullivan 2020). The propositions available are as follows: to not conduct animal testing and instead proceed with herd immunity; to conduct in-vitro human clinical trials; or to consider the ethics of animal testing. The answer is not straightforward because of the contradiction between animals as kin and animal exploitation. One way forward to address this contradiction is to consider Braidotti’s (2019: 182) statement: ‘“We”-who-are-not-one-and-the-same-but-are-in-this-convergence-together’. By doing so, we can rethink our relationship with animals through morals and values. Morals and values force us to consider whether we inflict pain and suffering on laboratory animals to save human suffering, or treat these animals as kin and not exploit them.

Returning to the discussion about the Anthropocene, ‘“We”-who-are-not-one-and-the-same-but-are-in-this-convergence-together’ (Braidotti 2019: 182), means both humans and the more-than-human world (including animals) are vulnerable from the effects of the Anthropocene. Here is it useful to turn to Haraway’s discussion on staying with the trouble. This idea seems particularly pertinent at present:

In urgent times, many of us are tempted to address trouble in terms of making an imagined future safe, of stopping something from happening that looms in the future, of clearing away the present and the past in order to make futures for coming generations. Staying with the trouble does not require such a relationship to times called the future. In fact, staying with the trouble requires learning to be truly present, not as a vanishing pivot between awful or edenic pasts and apocalyptic or salvific futures, but as mortal critters entwined in myriad unfinished configurations of places, times, matters, meanings (Haraway 2016: 1).

Staying with the trouble is a way of taking moral responsibility: humans and the more-than-human world remain in the present, with the knowledge that we live in difficult times. It is not about imaginary safe futures or preventing something from happening. Staying with the trouble means ‘“We”-who-are-not-one-and-the-same-but-are-in-this-convergence-together’ (Braidotti 2019: 182) need each another in unforeseen collaborations and associations. The Covid-19 pandemic is forcing us to stay with the trouble. With no vaccine and no drugs currently able to help alleviate symptoms, strict lockdown measures across the world are the only method available to attempt to supress the virus. Out of necessity we have had to remain in the present. This is very much an example of staying with the trouble. At present, humans and Covid-19 are entangled, and staying with the trouble is the only approach that can be used to untangle these interwoven lives. By staying with the trouble we are acting responsibly or as Haraway (2016) would describe it, we are enacting response-ability. Response-ability is about ‘absence and presence, killing and nurturing, living and dying’ (Haraway 2016: 28). Now more than ever, we need to enact response-ability. We need to stay with the trouble and work out our way forwards in resolving how to deal with the Covid-19 pandemic. By being present in the situation, we can find solutions which not only assist with the Covid-19 pandemic but also deal with the situation which led us to this problem in the first place. That problem was exploiting animals and their environment. Basically, humans need to change their ways, as up until this point, we have not done a good job of staying with the trouble or enacting response-ability.

Whatever happens with Covid-19, our lives will not be the same again. The Anthropocene is an important consideration in our argument because of the human neglect of the entanglements of life. In the twenty-first century, we have seen outbreaks of SARS, bird flu, MERS, Ebola, and Nipah, and Covid-19 is just the latest of these diseases. It is unlikely to be the last if we do not rethink what our futures are.

By rethinking our future, both Haraway (2015) and Wark (2015) argue there is a temptation to reject the term Anthropocene because it is too anthropocentric. This is because ‘the ethics (a sense of care for and responsibility toward others) and the politics (a sense of relationality within and among groups) are still fundamentally human’ (Nappi and Wark 2019: 106). This is a sentiment we need to agree with in the light of Covid-19. Our gaze has for too long been focused on humanity and we have neglected to address the more-than-human world. Wark’s (2015: 224–225) suggestions for new terms to replace the Anthropocene going forward include *empirio-monism*, *proletkult*, a tektology, or *utopia* of *Red Star*. The point of these terms is to better address the problems we are currently living with and to move forwards towards a better world. In other words, humans need to organise differently. Whichever term we chose, one thing is clear. There needs to be a collective effort to address the problems the world faces.

Büscher and Fletcher (2020) and Bonneuil and Fressoz (2017) call for a move away from current dominant thinking in our ways of addressing issues such as climate change and biodiversity loss, and instead call for scientists, social scientists, practitioners, and stakeholders to come together and use their collective thoughts. However, we are still thinking in anthropocentric terms. Humans are organising differently but perhaps not in a radical enough way. ‘What we need then is an alternative realism. One which sticks close to the collaborative labours of knowing and doing. One which opens towards plural narratives about how history can work out otherwise. A realism formed by past experience, but not confined to it’ (Wark 2015: xxi). I suggest a realism which includes the more-than-human world, and one in which we stay with the trouble and we enact response-ability.

## Data and the National Health Service Covid-19 App

The final aspect to examine before trying to piece together all of the different entanglements is that of data and the role of digital technology. The UK’s contact-tracing app, the NHS Covid-19 app, is used as a case study. This particular app was chosen as I live in the UK, and this is the app I am most knowledgeable about. Before looking at the NHS Covid-19 app, it is important to discuss other apps which are being used throughout the world. According to Amnesty International (2020a), as of 26 May 2020, there are 45 countries which are using or intending to use a Covid-19 contact-tracing app. Table 1 provides a comparison of digital contact-tracing apps for some of these countries.

**Table 1:** Comparison of digital contact-tracing apps for various countries. Adapted from Ada Lovelace Institute (2020: 25) and Privacy International (2020)

Country	Voluntary or mandatory use	Data collected	Access to data	Covid-19 infection reporting	Contact alerting
UK	Voluntary	Creation of identifiers by nearby phones	User and public health authorities	Self-reporting and medical professionals	User and medical professionals
Singapore	Voluntary	Creation of identifiers by nearby phones	User only	Medical professionals	User and medical professionals
South Korea	Mandatory	Citizen location and credit card data	User and public health authorities	Self-reporting and medical professionals	User and medical professionals
Taiwan	Mandatory	Citizen location and credit card data	User and public health authorities	Self-reporting and medical professionals	User and medical professionals
France	Voluntary	Creation of identifiers by nearby phones	Under consideration	Under consideration	Under consideration
Estonia	Voluntary	Creation of identifiers by nearby phones	Under consideration	Under consideration	Under consideration
Other EU countries	Voluntary	Creation of identifiers by nearby phones	Under consideration	Under consideration	Under consideration
Israel	Mandatory	Citizen location and credit card data	User and public health authorities	Self-reporting and medical professionals	User and medical professionals
Colombia	Unknown	Personal data, details of ethnicity, and participation at protests	Unknown	Unknown	Unknown
Argentina	Voluntary	National Identification, email, and phone number	User	Unknown	Unknown
Poland	Voluntary	Reference photographs, phone numbers, and regular check-ins	Public health authorities, Polish Government, and user	Unknown	Unknown
Qatar	Mandatory	Name, National Identification, location data, and health status	User and public health authorities	Unknown	Unknown

Privacy International (2020) investigated Covid-19 health apps from several countries. They found that the Norwegian health app stores data for 30 days on a centralised server, and the Colombian app requests personal data and asks questions about ethnicity and participation at protests. They also found the Argentina app for self-diagnosis of Covid-19 requires people to provide their National Identification, email, and phone number, whilst the Home Quarantining app developed by the Polish government requires reference photographs, phone numbers, and regular check-ins. With the Polish Home Quarantining app, photographs are verified using facial recognition software and location data to ensure people are not violating quarantine orders (Amnesty International 2020b). Amnesty International’s Security Lab (Amnesty International 2020a) has been investigating Qatar’s contact-tracing app. The app is mandatory and people could face 3 years in prison if they refuse to use it. The investigation revealed security concerns which could have allowed cyber attackers and hackers to access a user’s personal information including name, national identification, location data, and health status. The app’s central server did not have any security measures in place to prevent data breaches occurring.

Amnesty International (2020a) has called for contact-tracing apps to be designed which incorporate privacy and data protection. Initiatives by Google and Apple, the European PEPP-PT initiative, the multi-institution DP-3T proposal, and MIT’s Safe Paths are all focused on privacy-preserving approaches to data collection (Ada Lovelace Institute 2020). Governments are able to build contact-tracing apps in parallel with these protocols.

However, there are problems with contact-tracing apps. As more citizens use contact-tracing apps, large amounts of personal data are being collected, used, and shared. Certain contact-tracing apps are using algorithms which are based on biased data, further discriminating certain groups (Dubov and Shoptaw 2020). Additionally, Amnesty International (2020b) is concerned that contact-tracing apps may endure beyond the pandemic. These contact-tracing apps could potentially form the basis of surveillance in a post-pandemic world.

In order to look at these problems in greater detail, attention now turns to the UK’s NHS Covid-19 app. At the time of writing (15 June 2020), the app is currently being trialled on the Isle of Wight. If the trial is successful, it will be rolled out nationwide. The NHS Covid-19 app works in the following way:

If you develop systems of coronavirus, the app will:

- anonymously warn other app users who have been near you
- provide advice from the NHS on the right action to take to help stop the virus spreading further
- help you to get a swab test (NHS 2020a).

Further advice provided by the NHS explains:

The app is free and simple to use. Once you’ve downloaded the app, Bluetooth technology on your phone will record the distance between other phones that also have the app installed. If you become unwell with symptoms of coronavirus, you can allow the app to inform the NHS. This will trigger a notification that the NHS will then send anonymously to all other app users who you’ve been in significant contact with over the previous few days. Affected app users will be sent official NHS advice on what to do next.

The app will be part of a wider approach that will involve contact tracing and testing for the virus (NHS 2020b).

As more people use the NHS Covid-19 app on their smartphones, more and more data will be collected about the virus and its spread throughout the community. But who is gaining from the use of the smartphone app? Here it is useful to consider ‘a politics of possibilities: ways of responsibly imagining and intervening in the configurations of power’ (Barad 2007: 246). Currently, although it is *only* a virus, Covid-19 holds power as it has drastically changed our lives and continues to do so. But does the use of the contact-tracing app transfer power to the UK Government and scientists through data collection and analysis of the data? Or does power transfer to those citizens who decide to use it in an effort to keep themselves safe? These are questions which will need to be addressed going forwards. But they show how including data in an entanglement between humans and a virus suddenly adds political dimensions.

What the NHS Covid-19 app does illustrate is a point made by Lupton (2020), in that there is potential for humans and digital data to learn from one another as they come together. As the NHS Covid-19 app is rolled out, more people will be using it. The NHS Covid-19 app learns from data analysis. It is through this data collection and analysis that humans and data learn from one another. The more interactions there are with people with coronavirus, the more data will be collected and the more Covid-19 will be understood.

If we dig deeper, there is more to the NHS Covid-19 app than just collecting data. Digital technologies are becoming increasingly powerful in terms of decision-making and policy making, but they are also becoming powerful in the understanding of our own identities (Thornham 2019). Lupton (2018) has examined programmes which have promoted patient self-care, preventive medicine, and health promotions, and she found narratives which foster gains for the individual alongside the community and the public good. This type of citizenship enables all to gain and is based on altruistic thinking. Certainly with the NHS Covid-19 app, people will need to be willing to share their data for others to gain. There is a narrative coming from the UK Government at present which is promoting the contact-tracing app in order for others to gain. As illustrated in a news interview, Matt Hancock, the Secretary of State for Health and Social Care, ‘suggested the public have a “duty” to install the contact-tracing app on their smartphones. “If you download the app you are doing your duty and you’re helping save lives” he told BBC Breakfast’ (The Guardian 2020). Here, a sense of duty shifts the onus of care onto citizens. Lupton (2018) has discussed the shifting responsibilities between doctor and patient, and argues that the digitally engaged patient becomes responsible for managing their own health when the responsibility of care is transferred from the clinician. The NHS Covid-19 app could be seen as an approach to encourage people to manage their own health and that of other people. The onus shifts from the UK Government tracking the spread of Covid-19, and instead relies on the data that is being collected by citizens and the NHS Covid-19 app.

If we are to use Haraway’s terminology of staying with the trouble, then we must acknowledge that problems are likely to arise with the NHS Covid-19 app. A range of characteristics including gender, ethnicity, race, religion, socio-economic status, location, and nationality determine how individuals become data subjects (Taylor 2017). These same characteristics will also work to introduce and reinforce inequalities, and discriminate and exclude certain groups (Dencik et al. 2019; Park and Humphry 2019). For example, an individual who is from the majority ethnic group is less likely to be targeted for surveillance by law enforcement (preventative surveillance) and social services (protective surveillance) than an individual who belongs to a minority ethnic group. Problems have already been noted with how data, algorithms, artificial intelligence, and machine learning can work to marginalise and discriminate against those in already marginalised groups (Eubanks 2018; Park and Humphry 2019). Contact-tracing apps have the potential to increase the magnitude and scale of issues which occur from problematic assumptions (Gillingham and Graham 2016). We need to recognise that not everyone has access to smartphones. However, this lack of access raises questions about this app and who it will protect. Those who are vulnerable in society are likely to be the most disadvantaged, as there is still a digital divide in the UK. In this context, ‘the internet “not knowing enough” rather than “knowing too much” becomes the data harm’ (Lupton 2020: 125).

The elderly, the vulnerable, and those living in poverty are most at risk from Covid-19, but these are the people least likely to have access to a smartphone. Does this mean that those who do not have access to this type of technology are at greater risk of catching coronavirus? If contact-tracing is only conducted by alerts from the NHS Covid-19 app, what will happen to those who are not carrying a phone? Will they be traced or will they fall through the net? These are all questions which require answering. Additionally, we are just at the beginning of the trialling and deployment of the NHS Covid-19 app and we do not know how many people will use it. We do not know if the app will be further developed to make it more sophisticated. What is important to recognise is that policy decisions made in the future may be based on the data collected. As Lupton (2020) argues, governments are increasingly using digital data to make policy decisions. For those social groups who do not interact with digital technologies, a lack of available data is likely to deepen socio-economic disadvantages. This is because these groups will not be incorporated into data-profiling activities. If policy decisions are made going forward based on data collected from the NHS Covid-19 app, then as we have seen, we face discriminating and marginalising those who may be at greatest risk from contracting the virus. This is especially pertinent if digital data is relied upon for resource and funding allocations.

Another issue relating to social justice is that contact-tracing apps are a form of surveillance or dataveillance. The NHS Covid-19 app will be generating information about those using the app, such as when a person comes into contact with someone who has Covid-19, as well as when a person records having symptoms of the virus. In this situation, in order for the app to function properly, dataveillance is a requirement. Whilst dataveillance can be a problem with data security and privacy, there is another problem relating to social justice. Certain social groups including the elderly, the very poor, the homeless, or those with disabilities who find it difficult to engage with digital technologies will be less exposed to dataveillance. This is due to them engaging less with digital devices and there being fewer interactions for data collection (Lupton 2018). These groups already tend to be marginalised and disadvantaged, so once again, we may exacerbate the disadvantages some already face. Here, disadvantages are created for individuals because they are *not* subjected to dataveillance.

It is also important to acknowledge that the NHS Covid-19 app raises questions about data security and privacy. A contact-tracing app is not a general purpose technology but a gatekeeping technology. When considering the uses of a contact-tracing app, it is necessary to think about whether it will be used for the advancement of human wellbeing or whether it will create significant risks for society (Leslie 2019). For my argument about data security and privacy, it is useful to examine digital healthcare technologies in general. Sharon (2018) coined the terminology, Googlisation of health research (GHR). This relates to how large datasets and Big Data are currently being used in health provision. There are concerns about data protection, privacy, and consent, but also concerns about the role technology companies play in healthcare and the new power relations that are being created between citizens, public health institutions, and technology companies. The concerns involving power relations relate to who have access to data, ownership of data, technical expertise, and technical infrastructure. All of these will shape healthcare.

Additionally, the digitalisation of healthcare is a highly lucrative business. Sixteen healthcare and biotech companies have earned the title of a ‘unicorn’ company. A unicorn company is a startup venture which reaches the $1 billion valuation mark without being quoted on the stock exchange. These include companies such as Babylon Health, Doctolib, and CMR Surgical (The Lancet Digital Health 2019). However, it is only through national and regional guidelines for the management of Big Data, algorithms, artificial intelligence, and machine learning will it be possible to establish what GHR can do (Sharon 2018). Currently though, there are inadequate policies to regulate Big Data, algorithms, artificial intelligence, and machine learning. This absence of regulation shows a lack of appreciation about how digital technologies are changing healthcare.

Healthcare technologies can improve the speed of diagnoses and improve efficiency and costs, but these technologies require better evaluation. When introducing technologies, they need to be done so in consultation with citizens. This enables citizens to consider and anticipate the potential consequences of Big Data, algorithms, artificial intelligence, and machine learning, as well as monitoring its use (Smallman 2019). By facilitating public engagement and allowing citizens to question how companies use data, there is an opportunity to build public trust (The Lancet Digital Health 2019). Public trust is important. Digital contact-tracing will only be effective if citizens trust it. Concerns about tracking movements are significant for some communities in the UK after incidents such as the London Metropolitan Police Service’s trial of facial recognition technology (Fussey and Murray 2019) and the London Metropolitan Police Service Gangs Violence Matrix (Amnesty International 2018). If communities lack trust in digital contact-tracing, they may suffer disproportionately from the effects of Covid-19. This in turn may mean Covid-19 spreads into wider society. In a pandemic situation, everyone needs to be protected equally.

Without building public trust, concerns can arise about data security and privacy. These anxieties about data security and privacy are often met with guarantees and assurances from the authorities, agencies, and organisations responsible for technologies and data collection that data is secure and will only be used for intended purposes (Iveson and Maalsen 2019). As a previous NHS example described by Moats and McFall (2019) illustrates, initiating public trust and maintaining it are important when digital technologies are introduced. Care.data^1^ was introduced by the NHS in 2014. Care.data collected data from primary care settings which included a patient’s NHS number, date of birth, postcode, gender, vaccination history, diagnoses, prescriptions, referrals, and family history. In January 2014, patients were informed about Care.data via a leaflet from NHS England. This leaflet was only produced after pressure from doctors, who wanted patients informed about how their highly personal information was to be used. Care.data gave patients the option to opt out of having their data sent to the Health and Social Care Information Centre (HSCIC), and over a million people did so within a few weeks. The problem arose here because the Department for Health did not anticipate the outrage patients would feel about their highly personal details being used by private companies. Patient data sharing was seen as an issue which could be dealt with through communication, opt-outs, and encryption, as opposed to building public trust. Due to its failure, Care.data was closed in 2016.

The case of Care.data illustrates what can happen if the introduction of digital technologies is not carefully considered. What the case also shows is that whilst digital connections can provide a company with a monopoly over data, a counter-movement can occur. In this case, citizens were wishing to protect their data. What happened in this situation is what Braidotti (2019) views as the positioning of different power relations. Power relations are an important consideration for data infrastructures. Data containing health information may provide a source of data for commercial companies ranging from medical research to the evaluation of health initiatives (Gillingham and Graham 2016). The power, politics, and interests which surround data infrastructures need to be questioned. It is important to engage with questions which query and probe who has access to data, who is developing the technologies which collect data, and who are we marginalising and excluding (Dencik et al. 2019; Iveson and Maalsen 2019).

## The Coming Together of Multispecies Entanglements with the Postdigital

‘The postdigital is hard to define; messy; unpredictable; digital and analog; technological and non-technological; biological and informational’ (Jandrić et al. 2018: 895). In the postdigital world, Covid-19 is part of this messiness. Covid-19 is biological because as a virus it affects both animals and humans; it requires social and cultural responses in order to address the problems it creates; and it is digital because some of the social and cultural responses require digital technology (Peters et al. 2020). But how does all this complexity fit together? Trying to explain all of the interactions with Covid-19 is not straightforward. Entanglements are not only constrained to those which are multispecies but also stretch out to the digital. This is where species and data meet and intra-act. The example of the NHS Covid-19 app illustrates how humans, Covid-19, and digital technology/data come together.

A useful concept to use in order to attempt to make sense of this tangling is that of assemblages. The concept of the *assemblage* is used as a way of ‘acknowledging the material and non-material, the human and the non-human, the fleshy and the ideational in ever-changing configurations’ (Lupton 2018: 12). Here, it is important to acknowledge that data is not autonomous. Data exists alongside humans and technologies. However, ‘one way to make sense of data is to think of them as the central concern of a complex sociotechnical assemblage. This data assemblage is composed of many apparatuses and elements that are thoroughly entwined, and develop and mutate over time and space. … Each apparatus and their elements frame what is possible, desirable and expected of data. Moreover, they interact with and shape each other through a contingent and complex web of multifaceted relations’ (Kitchin 2014: 24). When human bodies transect and interact with technologies, the concept of the cyborg can be used. This approach acknowledges the blurring, indistinct, and ambiguous boundaries between humans and technologies in the creation of assemblages (Haraway 2008; 2016; 2018). Lupton (2018) developed the concept, digital cyborg assemblages, to indicate the interactions between human actors and digital technologies. So now we have the concepts data assemblage and digital cyborg assemblages. Whilst these concepts are effective, they do fail to acknowledge interactions with the biological-more-than-human-world, specifically, in this case, Covid-19. When we have interactions between humans, other biological entities, and the digital, I argue that the concept *postdigital hybrid assemblage* may be usefully adopted.

Postdigital hybrid assemblages enable the coming together of humans, biological-more-than-human-worlds, and the digital. The concept enables us to see how the intra-actions through the coming together and entanglements between these three different entities transform society and nature as ‘informatics hybridises with biologies’ (Haraway 2018: 129). Postdigital hybrid assemblages enable us to push beyond boundaries, helping us understand Covid-19 and its impacts on society. However, postdigital hybrid assemblages are messy. This is because of the intra-actions that Barad (2003) describes. It is the entanglement of assemblages and the intra-actions which occur between these assemblages, which creates this messiness. Here, we are dealing with intra-actions between humans, biological-more-than-human-worlds, and the digital.

Haraway (2014) in her presentation of ‘Anthropocene, Capitalocene, Chthulucene: Staying with the Trouble’ spoke about humans ‘incapacity to think the world that is actually being lived. The inability to confront the consequences of the worlding that one is in fact engaged in, and the limiting and thinking to functionality. The limit of thinking to business as usual’. I have already discussed issues with the NHS Covid-19 app from a social justice perspective. Now is the time to move away from thinking of business as usual and instead start thinking about the intra-actions between humans, Covid-19, and the digital.

As the NHS Covid-19 app is only just being introduced, it does not have to be done so in a way that is business as usual. As Braidotti (2019: 112) argues, we need ‘to embrace the opportunities offered by the new technologies and steer them towards new forms of solidarity and democratic debate and dissent’. We can use this opportunity to reimagine and reshape our relationship with technology from a social justice perspective. Here, it would be necessary to recall our past relationships with technologies, whilst considering the responsibilities we have to our fellow humans and the more-than-human world moving forward. Deboleena Roy (2018), who is a biologist, is happy with the idea that biology can initiate social change and social justice. It is just necessary to understand what social changes or social justice we wish to enact and the approaches we wish to take. If we include the more-than-human world, this does not mean we exclude humans, but instead, we include all forms of life in our journey for social justice (Roy 2018). This is what Haraway (2015: 161) sees as ‘multispecies ecojustice’.

Technologies are becoming more prevalent in society, and not always before a societal debate and conversation have taken place concerning their introduction. Social justice and multispecies ecojustice can be implemented without the introduction of technology. In the case of the NHS Covid-19 app, it could be just as effective to have human contact tracers conducting all of the UK Covid-19 tracing work. Just because a technology is available does not mean it is necessarily the best approach for solving a problem. Technology is not always a silver-bullet. What is required is a societal conversation about how social justice and multispecies ecojustice should be implemented so that there is a fair and just world for all. The extent of the inclusion of technologies should be open to societal debate. Here, the postdigital hybrid assemblage could be used to ensure that social justice and multispecies ecojustice is enacted.

## Conclusion

To express ambivalence with contact-tracing apps is not to indulge in technophobia or technophilia. Contact-tracing apps provide citizens with a tool to assist public health officials during a global health crisis. However, for some citizens there is no choice, as the use of contact-tracing apps is mandatory in certain countries. Ambivalence also gives us a chance to establish how some groups in society may be stigmatised or disenfranchised by contact-tracing apps. As a postdigital hybrid assemblage, contact-tracing apps also allow us to see how humans can engage with other biological-more-than-human-entities. Whilst there is no vaccine or treatments for Covid-19, humans are going to have to live alongside our more-than-human counterpart. We just need to learn to stay with the trouble and not to be afraid to stand up to what we are unsure of. Covid-19 is certainly making us aware of a lack of knowledge about dealing with this disease. Maybe the contact-tracing apps will help us put together some of the pieces of the jigsaw. As Haraway (2016: 133) argues, ‘neither the critters nor the people could have existed or could endure without each other in ongoing, curious practices. Attached to ongoing pasts, they bring each other forward in thick presents and still possible futures; they stay with the trouble in speculative fabulation’. Covid-19 is unlikely to be the last pandemic we see. Learning from our multispecies entanglements with and alongside digital technologies will enable us to move forwards in the future in a more robust manner.

The Covid-19 pandemic has affected everyone around the world, and the consequences are likely to affect humanity (Jandrić 2020) and the more-than-human world for a long period of time. What is evident is that this pandemic is raising many questions. The first question I set out to answer was: How do humans, Covid-19, and Covid-19 contact-tracing apps meet and intra-act? In answering this question, I have illustrated the coming together of multispecies entanglements and data entanglements. The multispecies entanglements are fundamental components in the social experiment described above, and data entanglements are also playing a pivotal role. The second question I set out to answer was as follows: What are the social justice issues and problems associated with contact-tracing apps? Here, I have shown how contact-tracing apps may increase the vulnerability of those who are already vulnerable, reduce and weaken data security and privacy, and undermine trust in healthcare provision.

One thing is clear. Our world is messy, and in order to make sense of it, we need to stay with the trouble we are currently facing. The coming together of humans, Covid-19, and contact-tracing apps, I see as a postdigital hybrid assemblage. This postdigital hybrid assemblage may help keep humans safe whilst they try to live their lives with the Covid-19 pandemic raging on. Whether it does remains uncertain. What I do hope is that this discussion about the postdigital hybrid assemblage will contribute to discussions in the future about how we are living our lives and who and what we are living our lives with.

## Footnotes

1 See https://www.england.nhs.uk/2013/10/care-data/. Accessed 4 May 2020.
